# Optimal growth temperature is not an adequate proxy of microbial cold adaptation

**DOI:** 10.1093/ismeco/ycag300

**Published:** 2026-10-19

**Authors:** Peng Wu, Ying Wang, Sha Yi, Qianqian Zhang, Yilin Shu, Yan Liao, Liang Shen

**Affiliations:** College of Ecology and Environment, Anhui Normal University, Wuhu 241000, Anhui, China; College of Life Sciences, Anhui Normal University, Wuhu 241000, China; College of Life Sciences, Anhui Normal University, Wuhu 241000, China; College of Life Sciences, Anhui Normal University, Wuhu 241000, China; College of Life Sciences, Anhui Normal University, Wuhu 241000, China; College of Ecology and Environment, Anhui Normal University, Wuhu 241000, Anhui, China; School of Life Sciences, University of Nottingham, Nottingham NG7 2RD, United Kingdom; College of Ecology and Environment, Anhui Normal University, Wuhu 241000, Anhui, China; College of Life Sciences, Anhui Normal University, Wuhu 241000, China

**Keywords:** cold adaptation, minimum growth temperature, optimal growth temperature, database

## Abstract

Microbial thermal adaptation is commonly characterized using the minimum (T_min_), optimum (T_opt_), and maximum (T_max_) temperatures that define the thermal growth range of microorganisms; with T_opt_ been frequently used to compare microbial thermal characteristics. Although previous studies have shown that T_min_ and T_opt_ reflect distinct aspects of microbial thermal physiology, the generality and quantitative limits of this relationship across microbial diversity have not been systematically evaluated. Here, we constructed a dataset of 14 023 microbial growth-temperature records to examine the relationships among T_min_, T_opt_, and T_max_. While T_opt_ showed strong overall correlations with both T_max_ (*r* = 0.910, *R*^2^ = 0.827, *P_adj_* < .05) and T_min_ (*r* = 0.793, *R*^2^ = 0.628, *P_adj_* < .05), the strength of these relationships varied markedly across the temperature spectrum. Within the lower-temperature range, the association between T_opt_ and T_min_ became weak and statistically insignificant (*r* = 0.106, *P_adj_* = .200). Furthermore, within 10–30°C T_opt_ range, microorganisms sharing similar T_opt_ values frequently exhibited substantially different T_min_ values, indicating considerable variation in their capacity for growth at low temperatures despite comparable T_opt_. These findings demonstrate that T_opt_ and T_min_ provide complementary rather than interchangeable information about microbial thermal characteristics, and T_opt_ alone is insufficient to predict lower thermal growth limits. Our study provides the most comprehensive quantitative assessment to date of the relationship between microbial cardinal growth temperatures, defining the limitations of using T_opt_ to predict lower thermal growth limits and highlighting the importance of explicitly reporting T_min_ in ecological, evolutionary and taxonomic studies.

Cold environments, including Antarctica, the Arctic, high alpine ecosystems (eg, the Tibetan Plateau and the Alps), and the deep sea, constitute a major fraction of Earth’s biosphere [1]. Microorganisms, hereafter referring to Bacteria and Archaea, sustain essential ecological processes in these environments and play major roles in biogeochemical cycling, especially in carbon and nitrogen turnover [2]. Understanding how microorganisms adapt to low temperatures is therefore not only important for microbial taxonomy and physiology, but also central to predicting ecosystem response to climate and environmental change.

Microbial thermal characteristics are commonly described using three cardinal temperatures: the minimum (T_min_), optimum (T_opt_), and maximum (T_max_) growth temperatures. Among these, T_opt_ is frequently reported and commonly used to compare the thermal characteristics of microorganisms [3, 4]. Classical classifications of psychrophilic and psychrotolerant microorganisms have also relied substantially on optimum and maximum growth temperatures [5, 6]. Previous physiological and ecological studies have shown that T_min_ and T_opt_ can reflect distinct aspects of microbial thermal physiology and have questioned the use of T_opt_ alone as an indicator of low-temperature growth capacity [7, 8]. However, the generality and quantitative limits of the T_opt_-T_min_ relationship across broad microbial diversity have not been systematically evaluated on a large scale.

T_opt_ identifies the temperature at which growth rate is maximized under laboratory conditions, whereas T_min_ describes the lower boundary at which growth can be detected under the conditions tested. Microorganisms with similar T_opt_ values may therefore differ substantially in their ability to grow at low temperature. For example, *Methanococcoides burtonii* has a higher T_opt_ (23°C) than *Methanogenium frigidum* (15°C), yet grows faster within its native Antarctic lake environment [9]. This contrast illustrates that optimal growth temperature and low-temperature growth performance are not necessarily interchangeable characteristics and may respond differently to environmental conditions.

To determine how consistently T_opt_ reflects lower thermal growth limits, we constructed a microbial growth temperature database mainly from species descriptions published in the *International Journal of Systematic and Evolutionary Microbiology* (IJSEM) and supplemented by records from other journals (i.e. Antonie van-Leeuwenhoek), integrating automated text extraction and manual curation. This workflow yielded 14 023 records containing reported T_min_, T_opt_, and T_max_ values extracted from the original species descriptions (Supplementary Table S1; Fig. S1). Using this dataset, we addressed three questions: (i) how T_opt_ relates overall to T_min_ and T_max_; (ii) whether the strength of these relationships remains consistent across the full thermal spectrum; and (iii) to what extent T_opt_ distinguishes microorganisms with different lower thermal growth limits.

Across all 14 023 records, T_opt_ showed strong overall correlations with both T_max_ and T_min_, although these relationships were not equally stable, with T_opt_–T_max_ association (*r* = 0.910, *R^2^* = 0.827) consistently stronger than T_opt_–T_min_ association (*r* = 0.793, *R^2^* = 0.628) (Fig. 1A). More importantly, sliding-window analyses showed that the strength of the T_opt_–T_min_ relationship varied markedly across the temperature spectrum. A clear transition in the T_opt_–T_min_ relationship was observed around 18–20°C (Fig. S2), and we therefore used 20°C as a conservative reference threshold for subsequent analyses. Below this temperature range, microorganisms sharing similar T_opt_ values frequently differed by several tens of degrees in their T_min_, particularly within the low-temperature range (Fig. 1B). For example, within the 15–20°C T_opt_ interval, reported T_min_ values ranged from −29.5 to 15.0°C, spanning more than 40°C, with similarly broad variation observed in the 20–25°C and 25–30°C intervals (Fig. 1B, Table S2). Consistent with this pattern, local correlation analyses further showed that T_opt_–T_min_ relationship became weak and frequently statistically insignificant within the lower-temperature range (Fig. 1C & D). Window-specific significance tests further showed that the T_opt_–T_min_ association frequently lost statistical significance below 20°C, whereas the T_opt_–T_max_ relationship remained significant across most temperature windows (Fig. 1E).

**Figure 1:**
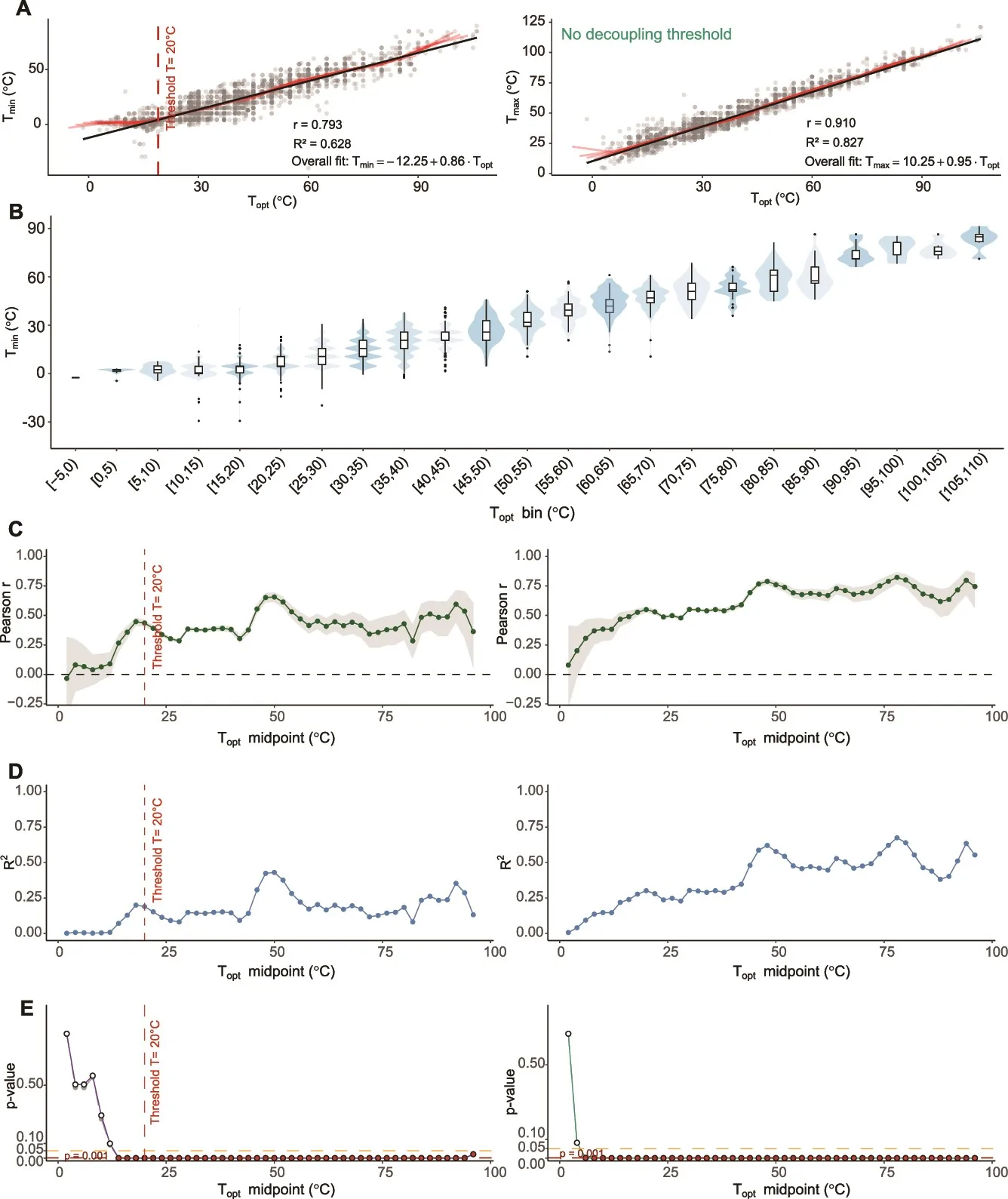
Global and local relationships between T_opt_ and T_min_, T_opt_, and T_max_. A. Raw scatterplots showing global and window-specific relationships between T_opt_–T_min_ and T_opt_–T_max_. Sliding windows were 15°C wide with a 2°C step, yielding 48 valid windows for both the T_min_ (left) and T_max_ (right) analyses. B. Distribution of T_min_ across 5°C T_opt_ bins. Violin plots show the overall distribution of T_min_ within each T_opt_ bin, and boxplots show medians and interquartile ranges. C. Local Pearson correlation coefficients (*r*) across sliding windows. Dark green lines and points show Pearson’s *r* for each sliding window, and gray bands indicate 95% confidence intervals. D. Local coefficients of determination (*R^2^*) from window-specific linear models. Blue lines and points show the *R^2^* values of the window-specific linear models. E. Raw and Benjamini-Hochberg (BH)-adjusted *P* values for local correlation tests. Filled red points indicate adjusted *P* < .05, whereas open points indicate non-significant windows.

Our results quantitatively support previous physiological observations that T_min_ and T_opt_ reflect distinct aspects of microbial thermal physiology while demonstrating that this relationship is not uniform across the full microbial temperature spectrum. Microorganisms with similar optimal growth temperatures can exhibit substantially different lower thermal growth limits, indicating that T_opt_ and T_min_ provide complementary rather than interchangeable information about microbial thermal characteristics. Consequently, T_opt_ alone has limited value for distinguishing microorganisms with different capacities for growth at low temperatures.

These findings have important implications for microbial ecology and the training of AI models to predict microbial growth under cold conditions. In permanently or seasonally cold ecosystems, microorganisms rarely experience conditions supporting maximal growth. Instead, persistence depends on the ability to maintain growth and metabolism near the lower end of the temperature range [10]. From this perspective, reported T_min_ more accurately reflects ecological adaptation to cold environments than T_opt_, because it directly reflects the capacity for growth persistence under chronically cold conditions [11]. Our results therefore suggest that T_opt_ and T_min_ should be interpreted as complementary descriptors of microbial thermal characteristics rather than interchangeable indicators.

Our findings also have implications for how microbial cold adaptation is interpreted in physiology and taxonomy. Psychrophilic and psychrotolerant microorganisms are frequently classified or compared using T_opt_ as a primary physiological descriptor (Fig. S3). However, our results show that microorganisms with similar T_opt_ values may possess significantly lower growth boundaries, suggesting that reliance on T_opt_ alone may underestimate differences in lower thermal growth limits. In many published species descriptions, the reported growth range does not clearly distinguish between the lowest temperature tested, the lowest temperature permitting detectable growth, and the true physiological lower growth limit (Table 1). We therefore recommend that future species descriptions and physiological characterizations report T_min_, T_opt_, and T_max_ together, explicitly document the temperature range examined, and indicate whether growth remained detectable at the lowest temperature tested. Such reporting would improve the interpretation and comparability of microbial growth-temperature data across studies.

**Table 1:** Examples illustrating that the preset temperature gradient did not span the complete growth temperature range, and the growth occurred at the lowest tested temperature.

Strain	Preset temperature range	Growth occurring	References
*Chromohalobacter sarecensis* LV4^T^	0–50°C	0–45°C	[14]
*Pantoea phytostimulans* C3^T^	4–40°C	4–36°C	[15]
*Gillisia lutea* M10.2A^T^	4–42°C	4–37°C	[16]
*Shewanella septentrionalis* SP1W3^T^	4–42°C	4–42°C	[17]
*Planococcus notacanthi* APC 4016^T^	4–44 °C	4–40 °	[18]
*Lacrimispora brassicae* YZC6^T^	10–45°C	10–37°C	[19]
*Salipaludibacillus daqingensis* DQ-9^T^	10–55°C	10–40°C	[20]

In summary, building upon previous physiological studies [7], our large-scale quantitative analysis defines the quantitative limits of the relationship between T_opt_ and T_min_ across microbial diversity, especially in defining the cryospheric lineages [12, 13]. Rather than challenging the concept that these cardinal temperatures reflect different aspects of microbial thermal physiology [3], our results establish the extent to which T_opt_ can be used to infer lower thermal growth limits. More broadly, these findings highlight that lower thermal growth limits cannot always be inferred from T_opt_ alone. One possible explanation is that the ability to sustain growth at subzero or near-subzero growth temperatures represents an emergent physiological trait that cannot be predicted directly from optimal growth temperature [13]. The weakening of the T_opt_–T_min_ relationship in the lower-T_opt_ windows may also reflect the increasing importance of physiological constraints that are not directly captured by T_opt_. We hypothesize that variation in the capacity to synthesize or acquire cold-protective compounds (compatible solutes like trehalose and betaine) contributes to variation in T_min_ among microorganisms with similar T_opt_. If so, persistence near environmental extremes may represent a distinct and underappreciated dimension of microbial thermal adaptation. Rather than viewing microbial thermal adaptation through a single cardinal temperature, our findings support a multidimensional framework in which different cardinal temperatures capture complementary aspects of microbial thermal physiology.

## Supplementary Material

Supplementary_material_ycag300

## Data Availability

The complete process of data extraction and workflow are available on GitHub (https://github.com/WPwp1/IJSEM_OGT).

## References

[1] Sugden S, Davis CL, Quinn MW, et al. Current and projected effects of climate change in cryosphere microbial ecosystems. *Nat Rev Microbiol* 2026;24:239–54. 10.1038/s41579-025-01251-1 41193719

[2] Holmberg SM, Jørgensen NOG. Insights into abundance, adaptation and activity of prokaryotes in Arctic and Antarctic environments. *Polar Bio* 2023;46:381–96. 10.1007/s00300-023-03137-5

[3] Ratkowsky DA, Lowry RK, McMeekin TA, et al. Model for bacterial culture growth rate throughout the entire biokinetic temperature range. *J Bacteriol* 1983;154:1222–6. 10.1128/jb.154.3.1222-1226.1983 PMC217594 6853443

[4] Sauer DB, Wang D-N, Valencia A. Predicting the optimal growth temperatures of prokaryotes using only genome derived features. *Bioinformatics* 2019;35:3224–31. 10.1093/bioinformatics/btz059 PMC6748728 30689741

[5] Moyer CL, Morita RY. Psychrophiles and Psychrotrophs. Encyclopedia of Life Sciences 2007; 1:1–6. Retrieved from: 10.1002/9780470015902.a0000402.pub2

[6] Morita RY . Psychrophilic bacteria. *Bacteriol Rev* 1975;39:144–67. 10.1128/br.39.2.144-167.1975 PMC413900 1095004

[7] Russell NJ . Cold adaptation of microorganisms. *Philos Trans R Soc B* 1990;326:595–611. 10.1098/rstb.1990.0034 1969649

[8] Cavicchioli R . Cold-adapted archaea. *Nat Rev Microbiol* 2006;4:331–43. 10.1038/nrmicro1390 16715049

[9] Cavicchioli R . On the concept of a psychrophile. *ISME J* 2016;10:793–5. 10.1038/ismej.2015.160 PMC4796924 26371407

[10] Wood C, Magnuson E, Harrop E, et al. Subzero cell division, respiration, and genomic traits of cryophilic Arthrobacter agilis ant-EH-1 isolated from cold-arid Antarctic mineral soils. *Front Microbiol* 2025;16:1620620. 10.3389/fmicb.2025.1620620 PMC12602508 41229692

[11] Shen L, Liu Y, Allen MA, et al. Linking genomic and physiological characteristics of psychrophilic Arthrobacter to metagenomic data to explain global environmental distribution. *Microbiome* 2021;9:136. 10.1186/s40168-021-01084-z PMC8196931 34118971

[12] Lee K, Kim S-H, Moon S, et al. Phyletic patterns of bacterial growth temperature in Pseudomonas and Paenibacillus reveal gradual and sporadic evolution towards cold adaptation. *ISME Commun* 2024;4:ycae163. 10.1093/ismeco/ycae163 PMC11694702 39748804

[13] Shen L, Liu Y, Chen L, et al. Genomic basis of environmental adaptation in the widespread poly-extremophilic Exiguobacterium group. *ISME J* 2024;18:wrad020. 10.1093/ismejo/wrad020 PMC10837837 38365240

[14] Quillaguamán J, Delgado O, Mattiasson B, et al. *Chromohalobacter sarecensis* sp. nov., a psychrotolerant moderate halophile isolated from the saline Andean region of Bolivia. *Int J Syst Evol Micr* 2004;54:1921–6. 10.1099/ijs.0.63153-0 15545411

[15] Campillo-Brocal JC, Sánchez-García M, Malo-López-Román J, et al. *Pantoea phytostimulans* sp. nov., a novel strain with plant growth-promoting properties. *Int J Syst Evol Micr* 2025;75:006764. 10.1099/ijsem.0.006764 PMC12038178 40279255

[16] Vidal-Verdú À, Molina-Menor E, Pascual J, et al. *Gillisia lutea* sp. nov., isolated from marine aluminium residues from the Mediterranean Sea. *Int J Syst Evol Micr* 2023;73:005957. 10.1099/ijsem.0.005957 37565577

[17] Martín-Rodríguez AJ, Thorell K, Joffré E, et al. *Shewanella septentrionalis* sp. nov. and *Shewanella holmiensis* sp. nov., isolated from Baltic Sea water and sediments. *Int J Syst Evol Micr* 2023;73:005767. 10.1099/ijsem.0.005767 37083582

[18] Uniacke-Lowe S, Stanton C, Hill C, et al. *Planococcus notacanthi* sp. nov., isolated from the skin of a deep-sea snub-nosed spiny eel. *Int J Syst Evol Micr* 2024;74:006298. 10.1099/ijsem.0.006298 PMC10963906 38512752

[19] Kobayashi H, Tanizawa Y, Sakamoto M, et al. *Lacrimispora brassicae* sp. nov. isolated from fermented cabbage, and proposal of Clostridium indicum Gundawar et al. 2019 and Clostridium methoxybenzovorans Mechichi et al. 1999 as heterotypic synonyms of Lacrimispora amygdalina (Parshina et al. 2003) Haas and Blanchard 2020 and Lacrimispora indolis (McClung and McCoy 1957) Haas and Blanchard 2020, respectively. *Int J Syst Evol Micr* 2024;74:006456. 10.1099/ijsem.0.006456 PMC11316579 39016536

[20] Guo L, Liu J, Hou Q, et al. *Salipaludibacillus daqingensis* sp. nov., a moderately halophilic bacterium isolated from an oilfield. *Int J Syst Evol Micr* 2023;73:006126. 10.1099/ijsem.0.006126 37917134

