# Supplementary material for: Optimal growth temperature is not an adequate proxy of microbial cold adaptation
Source: ISME Commun. 2026 Oct 19;6(1):ycag300. doi: 10.1093/ismeco/ycag300 (PMC13723765; doi:10.1093/ismeco/ycag300)
Supplement: Supplementary file 1 [file supplementary_material_ycag300.zip › Supplementary_Materials-R1_ycag300.docx]

**Supplementary Materials**

**Optimal growth temperature is not an adequate proxy of microbial cold adaptation**

Peng Wu, Ying Wang, Sha Yi, Qianqian Zhang, Yilin Shu, Yan Liao, Liang Shen

# Contents

[**Supplementary Materials and Methods**	**2**](#_Toc234329626)

[Data sources and curation	2](#_Toc234329627)

[Taxonomic matching and dataset structure	2](#_Toc234329628)

[Statistical analyses	3](#_Toc234329630)

[Traditional psychrophile and psychrotroph subset analysis	4](#_Toc234329629)

[**Supplementary figures	6**](#_Toc234329631)

[1. Taxonomic structure of the 14,023-record dataset	6](#_Toc234329632)

[2. Data-driven identification of the transition in the Topt–Tmin relationship.	7](#_Toc234329633)

[3. Traditional psychrophile and psychrotroph definitions analysis.	8](#_Toc234329634)

[**Supplementary tables	9**](#_Toc234329635)

[1. 14,023 microbial growth-temperature dataset (provided in excel format).	9](#_Toc234329636)

[2. Dispersion of T_min_ within 5 °C T_opt_ bins.	9](#_Toc234329637)

# Supplementary Materials and Methods

## Data sources and curation

The final analysis was based on the manually curated Supplementary Table S1, which contains 14,023 microbial growth-temperature records. Each retained record includes a species label, strain information when available, and a complete triplet of cardinal growth temperature: minimum growth temperature (T_min_), optimal growth temperature (T_opt_), and maximum growth temperature (T_max_).

The temperature records were mainly compiled from open-access taxonomic literature. The core source was open-access articles published in the *International Journal of Systematic and Evolutionary Microbiology* (IJSEM) from 1971 to 2026. In total, we screened 15,453 IJSEM-related articles and also included a small number of records from other taxonomic journals, such as *Antonie van Leeuwenhoek*. After automated text extraction, data cleaning, and manual verification, we obtained 14,023 reliable microbial records containing all three cardinal growth temperatures: T_min_, T_opt_, and T_max_.

All temperature values were converted to numeric values in degrees Celsius. When the source article reported a growth-temperature range, the lower bound of the growth-positive range was recorded as T_min_ and the upper bound was recorded as T_max_. When the optimal growth temperature was reported as a range, the midpoint of that range was used as T_opt_ during data extraction and in the main analysis. Records were retained in Supplementary Table S1 only when all three cardinal temperatures were available and satisfied the relationship T_min_ < T_opt_ < T_max_.

## Taxonomic matching and dataset structure

To evaluate the taxonomic structure of the dataset, the final temperature table was matched against the GTDB-r232 taxonomy database. The matched records covered 43 phyla, 119 classes, 296 orders, 698 families. The proportions of records represented by the 15 most abundant families and genera are shown in Fig. S1A and Fig. S1B, respectively.

Because microbial growth-temperature traits may be phylogenetically structured, the 14,023 records should not be treated as fully independent evolutionary observations. We therefore further analyzed the T_opt_–T_min_ and T_opt_–T_max_ relationships within selected high-frequency phyla, families, and genera, especially those containing taxa capable of growth at low temperatures. These analyses were used to evaluate whether the low-temperature decoupling between T_opt_ and T_min_ was driven by a small number of overrepresented taxonomic groups. The results showed that the contrast between the T_opt_–T_min_ and T_opt_–T_max_ relationships was still observed across multiple major taxonomic groups (Fig. S1C & D). These results indicate that the dataset has broad taxonomic coverage and that the reduced ability of T_opt_ to resolve T_min_ at the low-temperature end is not dependent on a single dominant taxonomic group.

## Statistical analyses

We first used linear regressions to fit the global T_opt_–T_min_ and T_opt_–T_max_ relationships and calculated their global Pearson correlations (*r*) across all 14,023 records. We then used sliding-window analyses to evaluate how these relationships changed across different temperature ranges. The sliding-window width was set to 15 °C, with a 2 °C step. Within each window, Pearson’s correlation coefficient (*r*), coefficient of determination (*R²*), and the corresponding *P* value were calculated. *P* values were adjusted within each comparison using the Benjamini–Hochberg procedure.

To identify the temperature range where the T_opt_–T_min_ relationship began to weaken at the low-temperature end, we further performed a task-oriented breakpoint scan and bootstrap resampling (Fig. S2). For the breakpoint analysis, bilinear models were fitted across candidate breakpoints and compared using the Akaike information criterion (AIC). A candidate threshold was considered to satisfy the task-oriented criterion when the T_opt_–T_min_ relationship was weaker on the lower-temperature side of the breakpoint than on the higher-temperature side. Bootstrap resampling was then used to evaluate the stability of the estimated breakpoint. Based on these analyses, 20 °C was selected as a conservative reference threshold for subsequent statistical comparisons of the low-temperature range, including *r*, *R²*, and adjusted *P* values.

We further quantified the dispersion of T_min_ within 5 °C T_opt_ bins in Fig 1B (Supplementary Table 2). For each T_opt_ bin, we report the mean ± SD and observed range of Tmin, and the observed range of ΔT (Topt − Tmin). These statistics provide explicit quantitative support for the statement that similar T_opt_ values can correspond to substantially different lower thermal growth limits.

## Traditional psychrophile and psychrotroph subset analysis

We performed an extra analysis in our database using the classical physiological definitions. Under the traditional framework, psychrophiles are generally defined as microorganisms with an optimal growth temperature of approximately 15 °C or lower, a maximum growth temperature of approximately 20 °C or lower, and the ability to grow at 0 °C or below. Psychrotrophs are cold-tolerant bacteria or archaea that can grow at low temperature but have optimal and maximum growth temperatures above approximately 15 °C and 20 °C, respectively.

In the present study, we used the following operational definitions:

1. Psychrophile: T_opt_ ≤ 15 °C and T_max_ ≤ 20 °C.
2. Psychrotroph: T_opt_ ≤ 15 °C and T_max_ > 20 °C.

Using these criteria, the final dataset contained 104 psychrophile records and 135 psychrotroph records. This analysis was used as a sensitivity analysis rather than as the primary conceptual framework of the manuscript, because the traditional psychrophile/psychrotroph classification itself partly depends on T_opt_ and T_max_ (Fig. S3). Therefore, using these T_opt_/T_max_-defined categories as the main framework to test whether T_opt_ represents cold adaptation would introduce a degree of circular reasoning.

Within both subsets, the T_opt_–T_min_ relationships were weak and non-significant (Fig. S3A). In addition, the T_min_ distributions of psychrophiles and psychrotrophs overlapped broadly (Fig. S3B). These results indicate that, even within the classical low-temperature microbial categories, T_opt_ does not sufficiently resolve lower thermal growth limits. This supports the central argument of the manuscript: T_opt_ can describe maximal growth performance under laboratory conditions, but it should not be used alone to infer microbial cold adaptation or lower thermal growth boundaries.

# Supplementary figures

## Supplementary Figure 1. Taxonomic structure of the 14,023-record dataset and robustness analyses across representative families and genera.

Panels A and B show the distributions of the most represented families and genera, respectively. Panels C and D compare the T_opt_–T_min_ and T_opt_–T_max_ relationships within selected representative families and genera.

## Supplementary Figure 2. Data-driven identification of the low-temperature transition in the T_opt_–T_min_ relationship.

Panel A shows the task-oriented breakpoint scan. Each point represents the total AIC of a bilinear model fitted under a candidate breakpoint. Orange points indicate candidate thresholds satisfying the task-oriented criterion, namely that the T_opt_–T_min_ relationship is weaker on the lower-temperature side of the breakpoint than on the higher-temperature side. The shaded area indicates the support interval, and the dashed vertical line marks the optimal task-oriented threshold. This analysis identified a support interval of 18.5–20.0 °C, with 20.0 °C used as a conservative reference threshold. Panel B shows the bootstrap distribution of breakpoint estimates. The histogram represents the frequency distribution of breakpoint estimates obtained from bootstrap resampling. The solid vertical line indicates the bootstrap median threshold, and the dashed vertical lines indicate the 95% confidence interval. The bootstrap median breakpoint was 19.5 °C, with a 95% confidence interval of 17.9–20.0 °C.

## Supplementary Figure 3. Traditional psychrophile and psychrotroph definitions analysis.

Panel A shows linear fits for the T_opt_–T_min_ and T_opt_–T_max_ relationships within the psychrophile and psychrotroph subsets. Panel B shows the global distribution of the psychrophile and psychrotroph records in relation to T_opt_, T_min_, and T_max_, illustrating the broad overlap in lower thermal growth limits between these two operational categories.

# Supplementary tables

## Supplementary Table 1. Manually curated 14,023 microbial growth-temperature dataset (provided in excel format).

Supplementary Table 1 contains the 14,023 microbial growth-temperature records used in the main analysis. Each record includes the species label, strain information, T_min_, T_opt_, T_max_, source journal, article title, DOI or source identifier, and taxonomic information. This table is the primary dataset underlying all analyses in the manuscript.

## Supplementary Table 2. Dispersion of T_min_ within 5 °C T_opt_ bins.

| Topt bin | Tmin mean±SD | Tmin range | Topt-Tmin range ($\Delta T=T_{\mathrm{opt}}-T_{\min}$) |
| --- | --- | --- | --- |
| [10, 15) | 1.23±5.75 | -29.5 to 13.0 | 1.0 to 44.0 |
| [15, 20) | 2.43±4.79 | -29.5 to 17.0 | 0.5 to 45.0 |
| [20, 25) | 6.36±5.16 | -14.5 to 22 | 0.5 to 35.5 |
| [25, 30) | 11.18±6.32 | -20.0 to 28.0 | 0.5 to 45.0 |
